# Correlation between Visual Acuity, Inner Segment/Outer Segment Junction, and Cone Outer Segment Tips Line Integrity in Uveitic Macular Edema

**DOI:** 10.1155/2015/853728

**Published:** 2015-06-08

**Authors:** Paolo Tortorella, Enzo D'Ambrosio, Ludovico Iannetti, Federica De Marco, Maurizio La Cava

**Affiliations:** Chorioretinal Diagnostic Service UOC Ophthalmology C, Department of Ophthalmology, “Sapienza” University of Rome, Viale del Policlinico, 15500161 Rome, Italy

## Abstract

*Purpose*. To investigate the correlation between best-corrected visual acuity (BCVA), the foveal inner segment/outer segment (IS/OS) junction or ellipsoid portion of inner segment (EPIS/ellipsoid zone), and the cone outer segment tips (COST) line or interdigitation zone integrity in eyes with uveitic macular edema (ME). *Method*. A retrospective observational study involving all patients from January 2012 to December 2013 with uveitic ME was performed. All patients underwent BCVA using Snellen charts spectral-domain optical coherence tomography (SD-OCT) examination using Spectralis OCT (Heidelberg Engineering, Heidelberg, Germany). *Results*. Fifty-two eyes from 45 patients were included in this study. Multivariate analysis showed a negative correlation between BCVA and the central retinal subfield thickness (CST), the cystoid pattern of edema, and the interdigitation zone interruption. Univariate logistic analysis showed a strong correlation between the ellipsoid zone and the interdigitation zone integrity. *Conclusions*. The ellipsoid zone defect, the interdigitation zone interruption, and the CST are correlated with poor vision. Visual acuity is also strongly affected by the cystoid pattern. The interdigitation zone integrity appears to be the most important factor in the visual prognosis of uveitic ME.

## 1. Introduction

Macular edema (ME) is a typical, but nonspecific, complication of uveitis and occurs most frequently in those with vitreous involvement. ME is among the leading causes of decreased vision in patients with uveitis [1, 2].

In the literature, ME is described in intermediate uveitis (25–70%), anterior uveitis (20–26%), panuveitis (35%), and posterior uveitis (20%) and it can dramatically affect vision [3].

Acute retinal necrosis, birdshot chorioretinopathy, Adamantiades-Behçet's disease, juvenile idiopathic arthritis, and sarcoidosis are the most common uveitis entities associated with ME [4].

Optical coherence tomography (OCT) is a noncontact and noninvasive diagnostic technique, which is increasingly used for diagnosing macular pathology and evaluating the response to therapy [4–7]. OCT provides a fundamental contribution to the diagnosis, guidance, and treatment of retinal pathologies such as macular edema, macular holes, epiretinal membranes, central serous chorioretinopathy, and age-related macular degeneration [5, 6].

In previous studies [2, 4, 8, 9] OCT findings were used to describe the three different morphologic patterns of ME: diffuse macular edema (DME), cystoid macular edema (CME), and serous retinal detachment (SRD). CME consists of low-reflective intraretinal spaces, clearly defined and separated by thin, high-reflective retinal tissue [2]. CME is one of the most frequent complications of uveitis and causes both blindness and visual impairment (29% and 41%, resp.) in uveitic patients [10]. DME consists of increased macular thickness, small low-reflective areas with spongy appearance of the retinal layers and SRD consists of a neuroretinal layer separation from the retinal pigment epithelium (RPE) [2].

Spectral-domain OCT (SD-OCT), the new OCT generation, was introduced recently. It provides a higher resolution and image formation up to 100-fold faster than the conventional time-domain OCT [11]. SD-OCT is equipped with an automatic and time system that performs the average of multiple B scan frames of the same site, providing an improved image quality [8]. The ability of SD-OCT to create images of tissue morphology in situ and in real time has been termed “optical biopsy.” High-resolution cross-sectional OCT scans can assist detailed analysis and evaluation of retinal lesions [7].

The integrity of the outer retinal layers—and particularly the photoreceptor layer—has gained much interest because of its close correlation with visual function [12]. A number of studies have highlighted this important correlation, encouraging a detailed analysis of the external retinal layers [13–17].

On SD-OCT, the outer retina has four distinct hyperreflective lines, which represent the external limiting membrane (ELM), inner and outer segments of the photoreceptors (IS/OS) junction otherwise named ellipsoid portion of inner segment (EPIS/ellipsoid zone), the cone outer segment tips (COST) otherwise named interdigitation zone, referred to as the intermediate line or Verhoeff's membrane, and the RPE. The innermost ELM is formed by the back reflection of the zonulae adherentes that joins the inner segment to the Müller cells. The EPIS is thought to represent the boundary between the inner and outer segments of the photoreceptors and is localized between the ELM and the RPE histologically. The interdigitation zone represents the outer tip of the cones. The outermost RPE line separates the photoreceptors from Bruch's membrane and choriocapillaris [12].

The purpose of the present study was to investigate the correlation between the best-corrected visual acuity (BCVA), the foveal EPIS, and interdigitation zone integrity in eyes with uveitic ME.

## 2. Materials and Methods

A retrospective observational study was performed on all patients with uveitic ME from January 2012 to December 2013. Inclusion criteria were ME diagnosed ophthalmoscopically associated with any anatomical type of uveitis (anterior, intermediate, posterior, and diffuse).

Exclusion criteria were other coexisting ocular diseases limiting visual acuity (VA): amblyopia, cataract, optic atrophy, macular epiretinal membrane (ERM), macular hole, or central scars.

Informed consent was obtained from all patients involved in this research. The study was conducted in accordance with local and regional regulations, good clinical practice, and the tenets of the Declaration of Helsinki.

The BCVA using Snellen charts was performed. ME was diagnosed by clinical examination.

All patients underwent SD-OCT examination with Spectralis OCT (Heidelberg Engineering, Heidelberg, Germany). Raster scans (20 × 15 degrees) consisting of 37 high-resolution horizontal B-scans were performed. SD-OCT evaluation was performed by three ophthalmologists.

**Figure 1:**
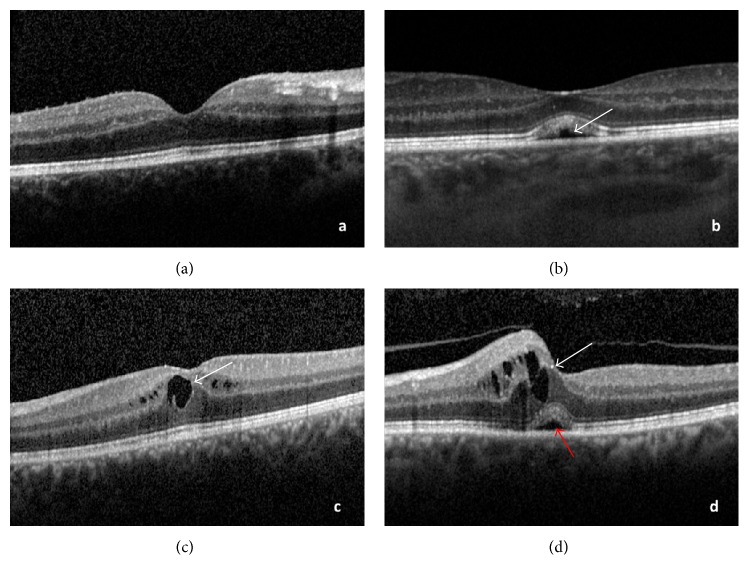
(a) Diffuse macular edema (DME). (b) DME associated with serous detachment of the neuroepithelium (SRD) (white arrow). (c) Cystoid macular edema (CME) (white arrow). (d) CME (white arrow) associated with SRD (red arrow).

Four patterns of ME, DME (Figure 1(a)), CME (Figure 1(c)), and SRD in combination with DME (Figure 1(b)) or with CME (Figure 1(d)), corresponding to a concentric zone of 1 mm in diameter around the fovea, were defined as reported previously [2].

**Figure 2:**
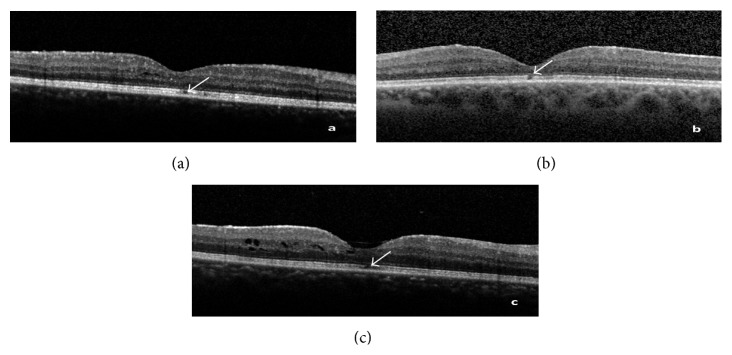
(a) Disruption of interdigitation zone. (b) Disruption of the EPIS. (c) Disruption of interdigitation zone and EPIS together (white arrows).

An evaluation of the central subfield retinal thickness (CST), the integrity or disruption of the interdigitation zone (Figure 2(a)), the integrity or disruption of the EPIS (Figure 2(b)), and the integrity or disruption of the interdigitation zone and the EPIS together (Figure 2(c)) was performed.

The CST measurement was considered a continuous variable and the mean ± SD was calculated. The decimal BCVA was measured on the Snellen decimal chart and considered ordinal data. As seen in the population distribution analysis of BCVA, the underlying population distribution did not exhibit a normal continuous distribution, even considering the BCVA above unity. Moreover, for retrospective studies, the truncation to 10/10 introduces a ceiling effect, making the normality assumption unsuitable (even asymptomatically) nor fixable using logMAR conversion. Thus, the median (1st–3rd quartile) values were reported. For the multivariate analysis, ordinal probit regression analysis (with CLM in the “ordinal” package) was used in the “*R* for statistical computing” environment, version 2.16 [18–20]. The correlations between the BCVA and CST, ME pattern, the integrity of the foveal EPIS, and the interdigitation zone were evaluated by multivariate correlation analysis. The full model was then evaluated by applying a stepwise procedure in both directions, automatically and manually, exploring interaction terms. The coefficients for the CLM model indicate the direction and strength of the effect of the covariate and were reported graphically, rather than numerically; + (−) was used to represent coefficients between 0 and 1 (−1) and ++ (− −) for coefficients >1 (<−1). *P* values were calculated using the Wald test. The CST in the multivariate analysis was intended to measure a 100 *μ*m increase in thickness. Univariate logistic regression analysis between the interdigitation zone interruption and the EPIS interruption was performed in *R* using GLM.

## 3. Results

Fifty-two eyes from 45 patients affected by uveitis, complicated by ME, with a median age of 32 years (*Q*_1_–*Q*_3_  9–77) were included in this study. The patients comprised 22 males and 23 females. Demographic and clinical characteristics of the study population are summarized in Table 1.

According to the site of inflammation, following the criteria of the Standardization of Uveitis Nomenclature [21], uveitis was classified as anterior in 12 eyes (23.07%), intermediate in 20 (38.46%), posterior in 7 (13.46%), and diffuse in 13 (25%) eyes.

The median duration of uveitis at examination was 48 months (*Q*_1_–*Q*_3_  2–204). The median BCVA was 0.6 (*Q*_1_–*Q*_3_  0.03–1.0). The mean (SD) CST was 411 *μ*m (±203).

SDOCT revealed DME in 39 eyes (75%), CME in 13 eyes (25%), and foveal SRD in 11 eyes (21.15%). SRD was found in combination with other forms of ME. In particular, three eyes (5.76%) presented SRD in combination with DME and eight eyes (15.38%) presented SRD in combination with CME.

The median BCVA in eyes with DME was 0.7 (*Q*_1_–*Q*_3_  0.03–1.0), that in eyes with CME was 0.6 (*Q*_1_–*Q*_3_  0.06–1.0), and that in eyes with foveal SRD was 0.4 (*Q*_1_–*Q*_3_  0.03–1.0).

Interruption of the EPIS was observed in 26 eyes (50%), and interruption of the interdigitation zone in 13 eyes (25%). The median BCVA in eyes with EPIS interruption was 0.35 (*Q*_1_–*Q*_3_  0.06–1.0) and it was 0.2 (*Q*_1_–*Q*_3_  0.06–0.8) in eyes with interdigitation zone interruption. The morphological features on SD-OCT with the corresponding CST and BCVA are reported in Table 2.

The multivariate regression showed a negative correlation between BCVA and CST (*P* = 0.0009), CME (*P* = 0.012), interdigitation zone interruption (*P* = 0.0005), and age (*P* = 0.003). The univariate logistic analysis showed a strong correlation between the EPIS segment and the interdigitation zone integrity (*P* = 0.04). In the multivariate analysis the EPIS or interdigitation zone interruption had the same effect and led to generation of comparable models (ANOVA LR *P* = 0.29).

## 4. Discussion

This study showed the correlations between VA and the cystoid pattern of ME, CST, EPIS, and interdigitation zone integrity in uveitic ME.

VA was strongly affected by the cystoid pattern (*P* = 0.012). High values of CST, which represent the increase in thickness and volume of the foveal area, negatively affect visual function (*P* = 0.0009). These results are in agreement with previous studies of ME secondary to uveitis [2, 8, 9].

In our study, the interdigitation zone interruption was the factor most significantly associated with poor vision (*P* = 0.0005). No previous study has investigated the correlation between VA and the interdigitation zone integrity in patients with uveitic ME. However, the importance of interdigitation zone integrity has been reported in other studies of various ocular diseases [13, 14, 22–24].

Ito et al. showed a strong correlation between VA and the status of the external limiting membrane (ELM), the status of the EPIS, and the status of the interdigitation zone in diabetic ME [13].

Shimozono et al. considered the status of the interdigitation zone, in conjunction with the EPIS, to be a useful prognostic factor after ERM surgery. The photoreceptor status at 1 month, especially the interdigitation zone, was the parameter most strongly correlated with the BCVA at 6 months after ERM surgery [14].

Itoh et al. reported a strong correlation between the interdigitation zone defect and the BCVA after pars plana vitrectomy for ERM removal. The interdigitation zone defect was significantly correlated with postoperative BCVA at 3, 6, 9, and 12 months, but not 1 month, postoperatively, suggesting continuous postoperative recovery from 1 to 12 months [22].

Itoh et al. investigated the correlation between the recovery of foveal cone microstructure and the BCVA after macular hole surgery. Eyes with an intact ELM and EPIS at 12 months and a distinct or irregular interdigitation zone had significantly better BCVA than those with a disrupted interdigitation zone [23]. Itoh et al. also showed that the length of the interdigitation zone defect was significantly correlated with VA at each postoperative timepoint. The integrity of the interdigitation zone, rather than the EPIS and ELM lines, may be a better clinical indicator of postoperative visual recovery in patients with surgically closed macular holes. They concluded that measurement of the preoperative length of the interdigitation zone may be an objective predictive factor of postoperative visual recovery [24].

It has been reported that disruption of the EPIS is associated with poor vision in uveitic ME [2].

Maheshwary et al. reported a significant negative correlation between VA and disruption of the EPIS in patients with diabetic ME. The rate of EPIS disruption evaluated by SD-OCT was revealed to be a significant predictor of VA [25].

In our previous study, EPIS disruption was strongly associated with CSF in uveitic ME but appears to be independent of the site of inflammation [2]. In this study, the differences among the groups were not statistically significant due to the small sample size.

Our statistical analysis showed a strong correlation between the EPIS and the interdigitation zone integrity (*P* = 0.04) which has not been reported previously in uveitic ME. EPIS disruption and the interdigitation zone defect, when considered together, showed a negative correlation with BCVA.

In conclusion, decreased vision has not been reported to be associated with the interdigitation zone defect in ME secondary to uveitis. Interdigitation zone integrity appears to be the most important factor in the visual prognosis of uveitic ME. An EPIS defect and interdigitation zone interruption and retinal thickness are correlated with poor vision. Also, the cystoid pattern affects VA. Further studies should investigate the prognostic role of the interdigitation zone in uveitic ME.

## Figures and Tables

**Table 1:** Demographic and clinical characteristics of the study population.

Median age (*Q*_1_–*Q*_3_)	32 (9–77)
Gender	
Male/female	22/23
Median FU mos (*Q*_1_–*Q*_3_)	25.5 (1–260)
Median duration (*Q*_1_–*Q*_3_)	48 (2–204)
Laterality	
Unilateral	38 (84.4%)
Bilateral	7 (15.6%)
Anatomic location of uveitis (eyes)	
Anterior	12 (23.1%)
Intermediate	20 (38.5%)
Posterior	7 (13.5%)
Panuveitis	13 (25%)
Classification of uveitis (patients)	
Idiopathic	36 (80%)
TBC	2 (4.4%)
Behçet	2 (4.4%)
VKH	2 (4.4%)
JIA	5 (11.1%)
Birdshot	1 (2.2%)
B27 + AAU	2 (4.4%)

**Table 2:** Number of eyes, mean foveal thickness, and median BCVA according to the different morphological features observed.

	*N* of eyes (% on 52 eyes)	Mean CST ± SD	Median BCVA (*Q*_1_–*Q*_3_)
DME	39 (75%)	354 ± 124	0.7 (0.03–1.0)
CME	13 (25%)	430 ± 222	0.6 (0.06–1.0)
SRD	11 (21.2%)	432 ± 154	0.4 (0.03–1.0)
No SRD	41 (78.8%)	406 ± 216	0.7 (0.06–1.0)
DME + SRD	3 (5.8%)	372 ± 176	0.7 (0.1–1.0)
CME + SRD	8 (15.4%)	467 ± 257	0.6 (0.1–1.0)
COST line disruption	13 (25%)	602 ± 303	0.2 (0.06–0.8)
COST line integrity	39 (75%)	348 ± 99	0.8 (0.03–1.0)
IS/OS junction disruption	26 (50%)	480 ± 253	0.35 (0.06–1.0)
IS/OS junction integrity	26 (50%)	342 ± 102	0.9 (0.03–1.0)
